# The relationships between three-axis accelerometer measures of physical activity and motor symptoms in patients with Parkinson’s disease: a single-center pilot study

**DOI:** 10.1186/s12883-020-01896-w

**Published:** 2020-09-10

**Authors:** Hiroto Ito, Daichi Yokoi, Rei Kobayashi, Hisashi Okada, Yasukazu Kajita, Satoshi Okuda

**Affiliations:** 1 Department of Neurology, National Hospital Organization Nagoya Medical Center, 4-1-1 Sannomaru, Naka-ku, Nagoya, Japan; 2 Department of Neurology, Kakeyu Misayama rehabilitation center, 1308 Kakeyuonsen, Ueda, Nagano, Japan; 3 Department of Neurosurgery, National Hospital Organization Nagoya Medical Center, 4-1-1 Sannomaru, Naka-ku, Nagoya, Aichi Japan

**Keywords:** Parkinson’s disease, Physical activity (PAL), Metabolic equivalents of task (METs), Three-axis accelerometer, UPDRS-3, Symptom diary, Wearable device

## Abstract

**Background:**

Various wearable devices for objectively evaluating motor symptoms of patients with Parkinson’s disease (PD) have been developed. Importantly, previous studies have suggested protective effects of physical activity in PD. However, the relationships between conventional clinical ratings for PD and three-axis accelerometer measures of physical activity (e.g., daily physical activity levels [PAL] or metabolic equivalents of task [METs]) are still unclear, particularly for METs. In the current study, we sought to elucidate these relationships on a daily basis, and to clarify optimal predictors for clinical states on a 30-min basis.

**Methods:**

Patients who were hospitalized for adjustment of drugs or deep brain stimulation were enrolled. Using waist-worn three-axis accelerometers, PAL and METs parameter data were obtained and compared with UPDRS-3[On] and symptom diary data. We extracted data from the patients’ best and worst days, defined by the best and worst UPDRS-3[On] scores, respectively. Thus, 22 data sets from 11 patients were extracted. We examined the correlations and produced scatter plots to represent the relationships, then investigated which METs parameters and activity patterns were the best predictors for “On” and “dyskinesia”.

**Results:**

The parameter “mean METs value within the 95–92.5 percentile range on a day (95–92.5 percentile value)” exhibited the strongest correlation with conventional daily clinical ratings (*Rho*: − 0.799 for UPDRS-3[On], 0.803 for On hours [*p* < 0.001]). Scatter plots suggested that PAL tended to have higher values in patients with involuntary movement. However, METs parameters focusing on higher METs seemed to alleviate this tendency. We clarified that “time over 2.0 METs” and “time over 1.5 METs” could be predictors for “On” and “dyskinesia” on a 30-min basis, respectively (AUROC: 0.779 and 0.959, 95% CI: 0.733–0.824 and 0.918–1.000). The specificity and sensitivity of the optimal activity pattern for “On” were 0.858 and 0.621.

**Conclusions:**

This study suggested feasible activity patterns and METs parameters for objective evaluation of motor symptoms on a 30-min or daily basis. Three-axis accelerometer measures focusing on higher METs may be appropriate for evaluating physical activity. Further larger-scale studies are necessary to clarify the validity, reliability, and clinical utility of these objective measures.

## Background

Parkinson’s disease (PD) is one of the most common age-related neurodegenerative disorders, characterized by various motor symptoms including slowness of movement (bradykinesia) as a main symptom, tremor (particularly at rest), and rigidity [1]. Although some PD symptoms are alleviated by medication and deep brain stimulation (DBS) [2], the effects gradually wear off as the disease progresses [1]. Motor symptoms become more complicated with fluctuation of symptoms (On-Off phenomena) and involuntary movement (dyskinesia) due to long-term use of levodopa [1]. Evaluation of various motor symptoms that gradually progress over time and change dynamically during the day is necessary in management of PD patients.

The current gold standard for evaluation of PD motor symptoms involves subjective evaluation for a certain period with a symptom diary [3, 4] and objective rating via doctor’s examination at specific time points in clinical settings (e.g., Movement Disorder Society Unified Parkinson Disease Rating Scale [UPDRS]-3) [5]. However, these subjective and objective methods have limitations related to low accuracy and poor temporal resolution, respectively [4, 6].

New methods and technologies using accelerometers or gyroscopes for the objective assessment of motor symptoms through different computational methods have been developed to overcome these limitations [6]. Previous assessments of motor symptoms in PD (such as bradykinesia or dyskinesia) have been attempted with systems such as the Parkinson’s KinetiGraph® system (PKG) [7] or Kinesia™ [8]. In some studies, however, contradictory results between UPDRS-3 (significant improvement) and measures of a wearable device (no significant improvement) have been reported [9, 10]. Although unobtrusive devices for patients are generally desirable for better compliance, some studies investigating the relationships between the gold standard and original scores have required PD patients to operate smartphones [11, 12]. These recently developed techniques have not yet become part of routine clinical assessment [6], and have not considered using the intensity of physical activity for objective assessment of motor symptoms.

In recent years, evidence suggesting the protective effects of physical activity against PD has accumulated [13]. A meta-analysis reported that the risk of developing PD was reduced in patients with higher daily physical activity levels (PAL) [14]. Animal experiments using PD model mice reported that exercise has protective effects against PD progression [15]. In chronic obstructive pulmonary disease (COPD), PAL reductions measured with a three-axis accelerometer were reported to be the greatest risk factor for death [16]. Metabolic equivalents of task (METs) are also considered to be a useful indicator of physical activity. One MET is the energy expended while sitting at rest. The METs for activity and exercise are calculated as a relative value to rest. Although the importance of maintaining PAL or METs measured with a three-axis accelerometer has been recognized in COPD [17, 18], few studies with wearable devices have focused on PAL [19, 20] or METs [21] in PD patients. Even in these studies, the potential influence of involuntary movement was not considered [19–21].

Thus, despite the potential utility of evaluating motor symptoms using three-axis accelerometer measures of physical activity and examining these measures in PD patients, their relationships with the conditions of motor symptoms are currently unclear, particularly for METs. Therefore, the current observational study sought to clarify the relationships on a daily basis between conventional clinical ratings (UPDRS-3 [On], On hours in symptom diary) and three-axis accelerometer measures (PAL and METs parameters [described in detail in *Methods*]) in various conditions among a variety of PD patients. Furthermore, we also investigated which METs parameters and activity patterns were the best predictors for the “On” and “dyskinesia” states on a 30-min basis.

## Methods

### Study population and design

Thirteen patients who were hospitalized for adjustment of drugs or DBS (patients were thought to exhibit variation in condition during hospitalization) were recruited from the Neurology Department at Nagoya Medical Center, Japan, between June 2017 and March 2018. Eligible participants had a diagnosis of idiopathic Parkinson’s disease. Other inclusion criteria were as follows: a) absence of severe cognitive impairment (Mini-Mental State Examination scores > 20); b) written consent was granted by the patients’ free will. Patients who met any of the following criteria were not included in the study: a) heart failure, COPD or acute diseases such as infectious disease that restrict exercise; b) the research director or research co-workers determined that participation in this study was not appropriate (such as a history of problematic behavior during hospitalization). We did not apply any cut-off points regarding physical activity or severity of PD to patients. Our study was conducted in a hospitalized setting to confirm patients’ conditions in more detail and in the same environment. The study protocol was approved by the ethical committee at Nagoya Medical Center. Written informed consent was obtained from all participants in accordance with the declaration of Helsinki.

### Objectives

Our primary objectives were as follows:

- Study I: To screen three-axis accelerometer measures (PAL and METs parameters [described in *Data analysis*]) to identify relationships (correlations, scatter plots) with conventional clinical ratings (UPDRS-3 [On] and On hours in symptom diary) on a daily basis.

We also investigated which three-axis accelerometer measures (among measures showing stronger correlations in study I) exhibited the largest change between the best and worst days.

- Study II: To investigate which specific METs parameters (described in *Data analysis)* are the best predictors of the “On” and “dyskinesia” clinical states on a 30-min basis.

In addition, we conducted activity pattern analysis to investigate the specificity and sensitivity of the defined activity patterns and their combinations (described in *Data analysis)* for “On”.

### Wearable device

A commercial three-axis accelerometer (Active Style Pro HJA 750C; OMRON, Kyoto, Japan [40 × 52 × 12 mm, 23.0 g]) was used for assessment of PAL and METs parameters [22]. The monitor collected three-axis acceleration data at 32 Hz, and METs data were recorded in 10-s epochs. The device was worn on the waist, and continuously monitored patients for more than 10 h per day. We referred to previous studies regarding this daily duration of assessment [23, 24]. Medical staff checked regularly to confirm the measurement period. The wearing time started between 07:00 a.m. and 07:30 a.m., and finished between 05:30 p.m. and 07:00 p.m. If the installation of the device was not completed, data were excluded. Data outside the wearing time (00:00 a.m. – 07:00 a.m., 07:00 p.m.– 00:00 p.m.) were also excluded. The time interval between monitoring ranged from 1 to 7 day(s) in DBS patients, and 7 days in drug adjustment patients.

### Conventional clinical ratings (UPDRS-3, on hours in symptom diary)

UPDRS-3 [On] [5] was evaluated 1–2 h after L-dopa administration [On]. In the symptom log, patients recorded On time (easy to move subjectively), intermediate time between On and Off, Off time (not easy to move subjectively), and the presence or absence of subjectively identified dyskinesia. We used the symptom diary data from 07:00 a.m. – 07:00 p.m. in study I, and the data from 07:30 a.m. – 05:30 p.m. in study II. Patients with dyskinesia were defined by the presence of dyskinesia clinically judged by the examiners. Patients with resting tremor were defined according to the sub-score of resting tremor in UPDRS-3 (sub-score ≥ 2).

### Approach

Because our protocol required hospitalization, we did not include healthy controls. Each data set (conventional clinical ratings and 3-axis accelerometer measures) was obtained on a daily basis. In drug adjustment patients, 2–3 opportunities (days) of evaluation were conducted on a daily basis before and after drug adjustment, and in DBS patients, 2–5 opportunities (days) on a daily basis were conducted for each setting of DBS. To reflect various conditions of patients and ensure the same number of data sets (2 days) in all patients, we extracted and analyzed data sets from the patients’ best and worst days, defined by UPDRS-3 [On] scores. Evaluation using conventional clinical ratings and three-axis accelerometer measures were also performed on the same days. Evaluation was stopped when the patient requested discontinuation, and data from those days were not included. Patients underwent rehabilitation as usual.

### Data analysis

METs determined by this three-axial accelerometer have been reported to be closely correlated with METs calculated using energy expenditure measured by indirect calorimetry [25, 26]. Basal metabolic rate (BMR) was estimated from a multiple regression equation including age, sex, height and ideal body weight as variables [27]. Total energy expenditure (TEE) was calculated by a manufactured regression equation using METs assessed by the triaxial accelerometer [26]. PAL was calculated by dividing TEE by BMR [28].

**Fig. 1:**
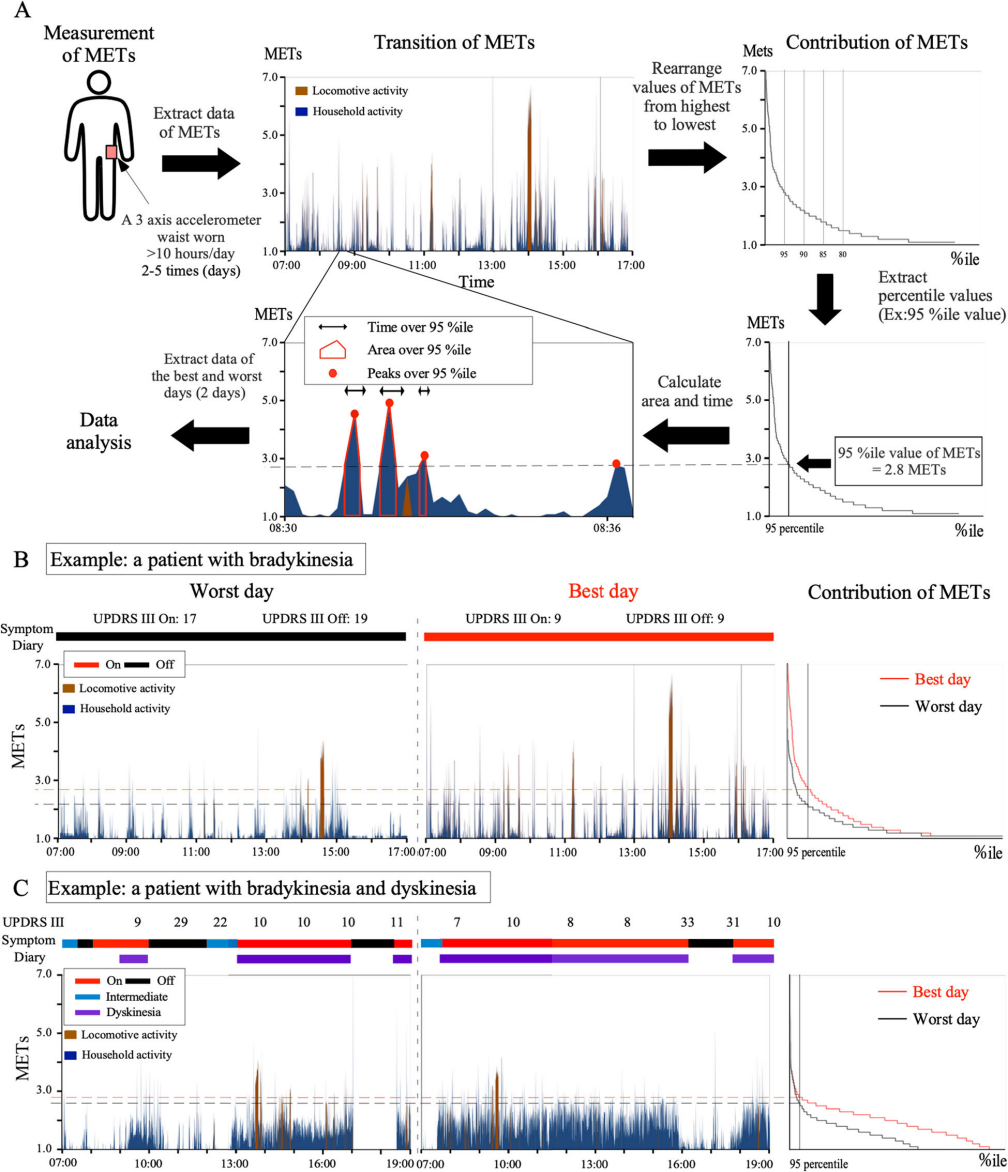
Data processing description and example results. **a** Data processing flow from measurement to analysis. Patients wore a three-axis accelerometer on the waist for more than 10 h per day and METs were measured every 10 s. METs data were extracted and lined up over time (transition of METs). METs data were then arranged from highest to lowest values (contribution of METs). Next, percentile values on a day were extracted (in this example case, the 95th percentile value was 2.8 METs). Finally, time, area and number of maximal values (peaks) over specific percentile values (in this example case, the 95th percentile value = 2.8 METs) and specific absolute METs values (e.g., 2 METs) were calculated. **b** Example results in a patient who had bradykinesia without involuntary movement (transition and contribution of METs, and 95 percentile value) are shown for the days on which the worst or best UPDRS-3 [On] ratings were observed. Transitions of METs appeared to be higher, longer, and more frequent on the patient’s best day compared with the patient’s worst day. **c** Example results of a patient with bradykinesia and involuntary movement (dyskinesia), suggesting a correlation between transition of METs with symptom diary states (“On” and “Off”) and the elevated baseline of METs (1.5–2.0 METs) during dyskinesia. The patient’s condition was checked every 1–2 h(s) objectively using UPDRS-3

The processing flow of the obtained METs data is described in Fig. 1a. METs data were extracted and lined up over time (Transition of METs). METs data were arranged from highest to lowest values (Contribution of METs). Then, each percentile METs value on a day against 12 h (the wearing time; 4320 epochs [1 epoch/10 s]) was extracted. Finally, the area/time/number of maximal values (peaks) over (≥) each percentile value (defined as the mean percentile value on the best and worst days in each patient) and specific METs values were calculated.

In study I, as METs parameters, each mean value within a range of 2.5 percentiles (from the 100th to 70th percentile) was calculated. Likewise, the area/time/peaks over specific METs values (every 0.5 METs from 1.5 to 3.0 METs) and percentile values (every 2.5 percentiles between the 100th and 90th percentile) were examined. In study II, area/time/peaks over specific METs value (every 0.5 METs from 1.5 to 2.5 METs) and percentile values (every 2.5 percentiles from the 100th to 90th percentile) on a 30-min basis were examined.

In the activity pattern analysis, the process of developing the definitions of criteria are described in Supplementary Fig. 3A–C. We defined criteria for activity patterns to distinguish “On” from “Non-On” as follows;

- Criterion I: at least one peak greater than 2.5 METs with four or more consecutive epochs over 2 METs.
- Criterion II: four or more peaks greater than 2.5 METs.

Continuous locomotive activity for more than 1 min is counted as just one peak.

- Criterion for “tremor”: “tremor” was determined by a combination of 90 epochs or more over 1.5 METs and clinical information of resting tremor (sub-score of UPDRS-3 ≥ 2). Tremor is included as “Non-On”.
- Exclusion criterion: we excluded peaks over 4 METs.

### Statistical analysis

As this pilot study was exploratory in nature, although no formal sample size estimation was performed, a sample of 11 participants was anticipated to be appropriate for a pilot study.

In study I, Spearman’s rank-order correlations were calculated between objective evaluation items measured with the three-axis accelerometer and conventional clinical ratings. We produced scatter plots of the primary results. Changes from the worst to best day of the three-axis accelerometer measures showing stronger correlations in study I were compared using *Z-*scores and nonparametric Wilcoxon signed-rank tests. In study II, the area under the receiver operating characteristic curve (AUROC) was used to assess model performance. In the analysis of “dyskinesia” in study II, the data from the only day on which the patient showed subjective dyskinesia were used in one patient. Statistical analysis was conducted using easy R version 1.40. The significance level was set to *p* < 0.05.

## Results

Eleven patients (mean age: 67.1 [56–81] years) completed repeated assessments more than twice during hospitalization. Ten patients provided data in symptom diaries (mean recorded time: 11.7 [10.0–12.0] hours). UPDRS-3 [On] and On hours were significantly lower and longer, respectively, on the best day compared with the worst day (mean ± *SD*: 18.6 ± 19.6 and 8.6 ± 5.1 [hours], 30.5 ± 20.0 and 1.8 ± 2.6 [hours], *p* < 0.01 and *p* = 0.013, Table 1). Other characteristics are shown in Table 1. Representative data (transition and contribution of METs on both the best and worst days) of patients with bradykinesia (Fig. 1b) and with bradykinesia and dyskinesia (Fig. 1c) are shown. In Fig. 1b, transitions of METs appeared to be higher, longer, and more frequent on the patient’s best day compared with the patient’s worst day. In Fig. 1c, an elevation of baseline METs (1.5–2.0 METs) was observed during dyskinesia.

**Table 1:** Clinical features of the included patients (*n* = 11)

Clinical feature: Mean (±*SD*) or Number (%)	
Age (years)	67.1 (± 7.7)
Mean disease duration (years)	13.8 (± 5.8)
Gender (%)	
Male	4 (36.4%)
Female	7 (63.6%)
Adjustment of Drugs/DBS (%)	
Drugs	3 (27.3%)
DBS	8 (72.7%)
MMSE	26.9 (± 2.7)
UPDRS-3 (On)	
Worst day	30.5 (± 20.0)
Best day	18.6 (± 19.6)
On hour(s)	
Worst day	1.8 (± 2.6)
Best day	8.6 (± 5.1)
Motor symptom (%)	
Bradykinesia	11 (100%)
Dyskinesia	3 (27.3%)
Resting tremor	4 (36.4%)
Sub-score of UPDRS-3 ≥ 2	2 (18.2%)
Postural instability	11 (100%)
Pulsion phenomenon	2 (18.2%)
Freezing of gait	3 (27.3%)
Physical activity (%)	
Sedatory	2 (18.2%)
Inactive	5 (45.5%)
Active	4 (36.4%)

*SD* Standard deviation, *DBS* Deep brain stimulation, *MMSE* Mini-Mental State Examination, *UPDRS* Unified Parkinson’s Disease Rating Scale. Sedentary: Usually uses a wheelchair. Inactive: Uses a wheelchair at times. Active: No use of a wheelchair

### Relationships between conventional clinical rating versus three-accelerometer measures on a daily basis in study I

**Fig. 2:**
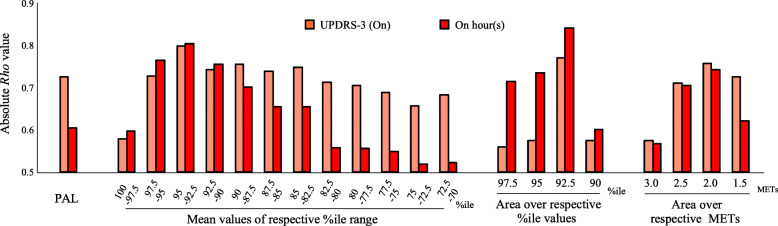
Correlation between conventional clinical rating and three-axis accelerometer measures on a daily basis. Main results for screening of absolute *Rho* values between three-axis accelerometer measures (each percentile value, PAL, area over percentile and absolute METs values) and conventional clinical ratings (UPDRS-3 [On] and On hour [s])

Twenty-two data sets were obtained from 11 patients. A stronger (anti) correlation was observed in the parameter “mean METs value within the 95-92.5 percentile range on a day (95–92.5 percentile value)” with both UPDRS-3 and On hours (*Rho*: − 0.799 [*p* < 0.0001] and 0.803 [*p* < 0.0001], Fig. 2) and “area over 92.5 percentile value” with On hours (*Rho*: − 0.841 [*p* < 0.0001], Fig. 2). All other screening results are summarized in Fig. 2 and Supplementary table 1. Regarding the parameter 95–92.5 percentile value, we also identified significant (anti-) correlations on both the best and worst day (*Rho*: − 0.797 [*p* < 0.01] and 0.682 [*p* < 0.05] with UPDRS-3; 0.716 and 0.710 [*p* < 0.05] with On hours), but only on the worst day for the parameter “area over 92.5 percentile value” (0.623 [*p* = 0.054] and 0.691 [*p* < 0.05] with On hours).

**Fig. 3:**
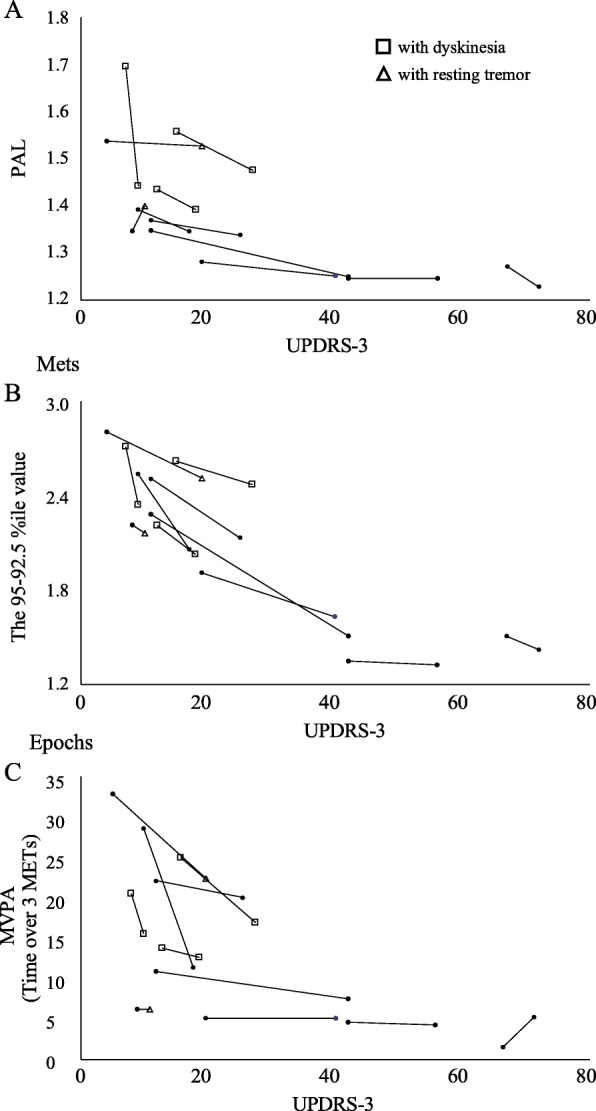
Scatter plots between conventional clinical rating and three-axis accelerometer measures on a daily basis. **a**–**c** Scatter plots for UPDRS-3 [On] and three-axis accelerometer measures (PAL, “mean METs value within the 95–92.5 percentile range on a day [95–92.5 percentile value]”, and “time over 3 METs [moderate to vigorous physical activity; MVPA]”). Plots of each patient’s worst and best days were connected with lines. **a** Plots of patients with involuntary movement (□ or △) were located among the higher values of PAL. The triangle (△) shows the day on which the patients had a sub-score of resting tremor in UPDRS-3 (sub-score ≥ 2). **b**-**c** By focusing on 95–92.5 percentile values or MVPA, the tendency for higher values in patients exhibiting involuntary movement appeared to be alleviated

We then created scatter plots (UPDRS-3 vs PAL, 95–92.5 percentile value, and time over 3.0 METs). Plots of patients exhibiting involuntary movement were located among the higher values of PAL in Fig. 3a. However, by focusing on 95–92.5 percentile value or time over 3.0 METs (considered to be moderate to vigorous physical activity [MVPA] [29]), the tendency for higher values in patients exhibiting involuntary movement appeared to be alleviated (Fig. 3b-c).

Next, we calculated the changes between the worst and best day in the measures showing stronger correlations in study I. Among them, the changes were largest in “area over 92.5 percentile value” (change of Z-score: 1.30, Supplementary Fig. 1).

### Three-accelerometer measures as predictors for “on” and “dyskinesia” clinical states on a 30-min basis in study II

The screening results for “On” and “dyskinesia” are summarized in Table 2 and Table 3, and Supplementary table 2A-B. Data were obtained from 10 patients (all patients with bradykinesia, two patients with resting tremor, and two patients with dyskinesia), with 392 and 398 segments (30-min basis) for the analysis of “On” and “dyskinesia”. The highest AUROC values for detection of “On” and “dyskinesia” were obtained in “time over 2 METs” and “time over 1.5 METs”, respectively (AUROC: 0.779 and 0.959, 95% CI: 0.733–0.824 and 0.918–1.000). The specificity and sensitivity of cut-off values (10 and 114 epochs) for these measures, determined by the smallest distance to the top-left corner of the ROC box, were obtained (0.639, and 0.770 for “On” and 0.960 and 0.912 for “dyskinesia”). The application of these cut-off values to each patient is shown in Supplementary Fig. 2A–B.

**Table 2:** Compariosn among METs parameters in predection for “On”

Parameters	AUROC	95% CI	*P*-value
Area			
Over 1.5 METs	0.747	0.698–0.795	< 0.001
Over 2 METs	0.773	0.727–0.819	0.030
Over 2.5 METs	0.752	0.705–0.800	0.033
Over 3.0 METs	0.673	0.621–0.725	< 0.001
Time			
Over 1.5 METs	0.740	0.692–0.789	< 0.001
Over 2 METs	0.779	0.733–0.824	reference
Over 2.5 METs	0.766	0.719–0.813	0.24
Over 3.0 METs	0.671	0.619–0.723	< 0.001
Peaks			
Over 1.5 METs	0.744	0.695–0.792	< 0.01
Over 2 METs	0.765	0.719–0.812	0.026
Over 2.5 METs	0.770	0.723–0.816	0.41
Over 3.0 METs	0.686	0.635–0.737	< 0.001

**Table 3:** Compariosn among METs parameters in predection for “dyskinesia”

Parameters	AUROC	95% CI	*P*-value
Area			
Over 1.5 METs	0.956	0.913–0.999	0.21
Over 2 METs	0.932	0.884–0.981	0.011
Over 2.5 METs	0.859	0.785–0.933	< 0.001
Over 3.0 METs	0.677	0.598–0.756	< 0.001
Time			
Over 1.5 METs	0.959	0.918–1.000	reference
Over 2 METs	0.943	0.897–0.989	0.062
Over 2.5 METs	0.872	0.799–0.946	< 0.01
Over 3.0 METs	0.678	0.595–0.760	< 0.001
Peaks			
Over 1.5 METs	0.942	0.881–1.000	0.16
Over 2 METs	0.930	0.862–0.998	0.041
Over 2.5 METs	0.870	0.782–0.957	0.010
Over 3.0 METs	0.713	0.622–0.804	< 0.001

*AUROC* Area under receiver operating curve, *CI* Confidence interval; *MET*, metabolic equivalents of task. (A) We compared the performance o f METs parameters as predictive models for the “On” state, with estimates of the area under the receiver operating characteristic curve (AUROC). The performance of time over 2 METs (AUROC: 0.779, 95% CI: 0.733–0.824) was higher than that of time and peaks over 2.5 METs and was significantly higher than the others. (B) We compared the performance of METs parameters as predictive models for the “dyskinesia” state, with estimates of AUROC. The performance of time over 1.5 METs (AUROC: 0.959, 95% CI: 0.918–1.000) was higer than that of area and peaks over 1.5 METs and time over 2 METs, and significantly higher than the others

Next, based on the results of study II, we developed criteria for activity patterns. The process of developing activity pattern criteria to distinguish “On” from “Non-On” is described in Supplementary Fig. 3A–C and their legends. The specificity and sensitivity of these defined activity patterns and their combinations are shown in Supplementary table 3 (0.858 and 0.621 in the optimal combination [criterion I or II, exclusion criterion and criterion for “tremor”]).

## Discussion

From study I, relatively high percentile values (e.g., 95–92.5 percentile value) may provide an objective severity measure for PD on a daily basis. The highest percentile values (e.g., 100–97.5 percentile range) were not strongly correlated with conventional ratings. This may be because some patients tended to have more peaks over relatively high METs values (≥4 METs), even on their worst day (Supplementary Fig. 3B), possibly due to loss of balance, or pulsion phenomenon. However, these values may not reflect the severity of PD. The lower percentile values (such as 85–70 percentile range) were not strongly correlated. This may be because lower METs increased in patients exhibiting involuntary movement compared with patients exhibiting only bradykinesia (Supplementary Fig. 2B).

Our results also suggest that involuntary movements in PD patients may increase PAL. This may be due to the elevated baseline of METs. Although several previous studies have investigated PAL using wearable devices, previous studies did not consider the influence of involuntary movement [19, 20]. The current results suggest that direct application of PAL measured with a three-axis accelerometer is not suitable for evaluating physical activity. Duration of physical activity over higher METs values (e.g., MVPA [29]) may be appropriate for evaluating physical activity in PD patients .

In the activity pattern analysis, we proposed activity patterns corresponding to “On”, “Non-On”, “tremor” and “dyskinesia” (Supplementary Fig. 3B). Higher, longer or more frequent increases in METs values may be explained by improvement of PD symptoms (slowness and less frequency of voluntary movement [1]), and lead to high performance of “95–92.5 percentile value” and “time over 2 METs”. The elevated baseline of METs in involuntary movement, “dyskinesia” was in accord with the high performance of “time over 1.5 METs”.

To the best of our knowledge, the current study is the first to use a commercially available three-axis accelerometer to investigate the relationships between conventional clinical ratings and METs parameters. The requirements for patients were simple; wearing the device only on the waist. This compact device may be feasible for clinical applications. For instance, it may be possible to objectively rate the gradual progression of disease severity by “95–92.5 percentile value” or the state on a 30-min basis by the defined activity pattern.

One limitation was the lack of a thorough evaluation in a daily life environment which is necessary to confirm the appropriateness of the device for outpatients. As for other limitations, we did not monitor the patients during the night period and for a more extended period of time (e.g., more than 3 days). Moreover, possible artefacts were not excluded, since we could not deny the possibility that higher METs might be caused by some activities, such as loss of balance or pulsion phenomenon. The results should be interpreted with caution because of the small sample size (particularly, a small number of patients with dyskinesia), and the absence of a healthy control group. Since we did not collect “tremor” state in the symptom diary, it is currently unclear whether dyskinesia is distinguishable from tremor based on only the data from our device.

## Conclusions

In study I, 95–92.5 percentile value exhibited the strongest correlation with conventional daily clinical ratings. Scatter plots suggested that objective measures focusing on higher METs (e.g., MVPA) may be appropriate for evaluating physical activity. In study II, the optimal predictors for “On” and “dyskinesia” were “time over 2.0 METs” and “time over 1.5 METs”, respectively. The defined activity patterns may be feasible predictors for “On”. Further larger-scale studies are necessary to clarify the validity, reliability, and clinical utility of these objective measures.

## Supplementary information

**Additional file 1: Figure S1.** Changes between worst and best day of three-axis accelerometer measures. †; *p* < 0.01, ‡; *p* < 0.001. Every measure was standardized using Z-scores, then compared. In the three axis accelerometer measures showing stronger correlations in study I, the largest changes were observed in “area over 92.5 percentile value (defined as the mean 92.5 percentile value on the best and worst days in each patient)”. The mean Z scores on the best and worst day are connected with a red line.

**Additional file 2: Figure S2.** Dot-plots of “time over 1.5 and 2 METs” and application of their cut-off values. Int, intermediate; Pt, patient. (A) Data were obtained from 10 patients, with 392 segments (dots). As a candidate for a cut-off value of “time over 2.0 METs”, 10 (epochs) were considered (red dashed line). (B) Data were obtained from 10 patients (six patients with bradykinesia, two patients with resting tremor, and two patients with dyskinesia), with 398 segments (dots). We considered 114 (epochs) as a candidate for the cut-off value of “time over 1.5 METs” (blue dashed line). Supplementary fig. 2B showed that patients with resting tremor also had values over the cut-off values. The application of the cut-off value for patient 11 (a patient with dyskinesia, who could not record a symptom diary) suggests increased dyskinesia time on the best day.

**Additional file 3: Figure S3.** Production process and criteria of activity patterns to distinguish “On” from “Non-On”. (A) ROC curve of “time over 2 METs” and “peaks over 2.5 METs”. To develop the criteria, we focused on the better predictors “time over 2 METs” and “peaks over 2.5 METs” for “On” (Tables 2 and 3). The cut-off values of these measures determined by the smallest distance to the top-left corner of the ROC box were 10 and 3 epochs, respectively. From these cut-off values, we calculated four (> 10 epochs/3 epochs = 3.3) or more consecutive epochs over 2 METs in Criterion I and four or more peaks greater than 2.5 METs in Criterion II. (B) The percentage and number of peaks over 4 METs on the worst and best days. The percentage appeared to be low (mean percentage; 3.1 [1.2–6.2] and 2.0 [0.81–5.3], *p* = 0.12;mean number; 13.7 [7–28] and 12.6 [8–34], *p* = 0.51), and a relatively higher percentage and larger number were observed on the worst day, suggesting that some PD patients on the worst day tended to have more peaks over 4 METs. On the basis of these results, we developed the criteria for exclusion of peaks ≥4 METs. (C) Criteria for activity patterns to distinguish “On” from “Non-On”. The definitions of the criteria for “On”, “Non-On” and “tremor (resting tremor)” . The criteria for dyskinesia are also described for reference. Regarding “tremor” (the sub-score of resting tremor in UPDRS-3 ≥ 2), we focused on “time over 1.5 METs”. A cut-off value of 90 epochs was determined, because patients with resting tremor showed milder elevation of baseline of METs compared with dyskinesia (reference) and almost all patients with bradykinesia did not have values over 90 epochs (Supplementary Fig. 1B). “Tremor” was classified as “Non-On”, even when patients met the criteria for “On”.

**Additional file 4: Table S1.** All screening results in study I.

**Additional file 5: Table S2.** Comparing state prediction (On, dyskinesia) using three-axis accelerometer measures (percentile) on a 30-min basis with ROC curve analysis in study II.

**Additional file 6: Table S3.** Specificity and sensitivity of the defined activity patterns and their combination.

## Data Availability

The datasets used and analyzed during the current study are available from the corresponding author on reasonable request.

## Abbreviations

PD: Parkinson’s disease
DBS: Deep brain stimulation
PAL: Physical activity levels
MET(s): Metabolic equivalent(s) of task
UPDRS-3: Movement Disorder Society Unified Parkinson Disease Rating Scale part 3
MVPA: Moderate to vigorous physical activity
COPD: Chronic obstructive pulmonary disease
BMR: Basal metabolic rate
TEE: Total energy expenditure
AUROC: Area under the receiver operating characteristic curve
