# Supplementary figures and images for: The relationships between three-axis accelerometer measures of physical activity and motor symptoms in patients with Parkinson’s disease: a single-center pilot study
Source: BMC Neurol. 2020 Sep 10;20:340. doi: 10.1186/s12883-020-01896-w (PMC7488269; doi:10.1186/s12883-020-01896-w)

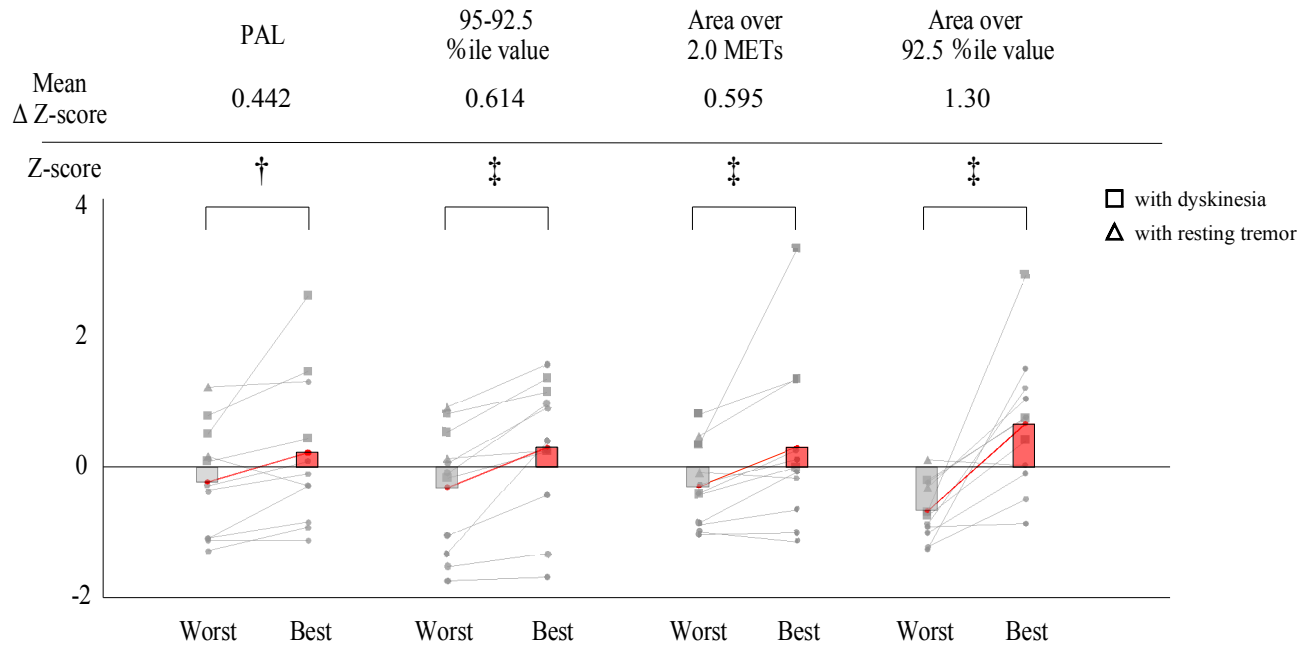

Supplement: Supplementary file 1 — Additional file 1: Figure S1. Changes between worst and best day of three-axis accelerometer measures. †; p < 0.01, ‡; p < 0.001. Every measure was standardized using Z-scores, then compared. In the three axis accelerometer measures showing stronger correlations in study I, the largest changes were observed in “area over 92.5 percentile value (defined as the mean 92.5 percentile value on the best and worst days in each patient)”. The mean Z scores on the best and worst day are connected with a red line. [file 12883_2020_1896_MOESM1_ESM.pdf]

A

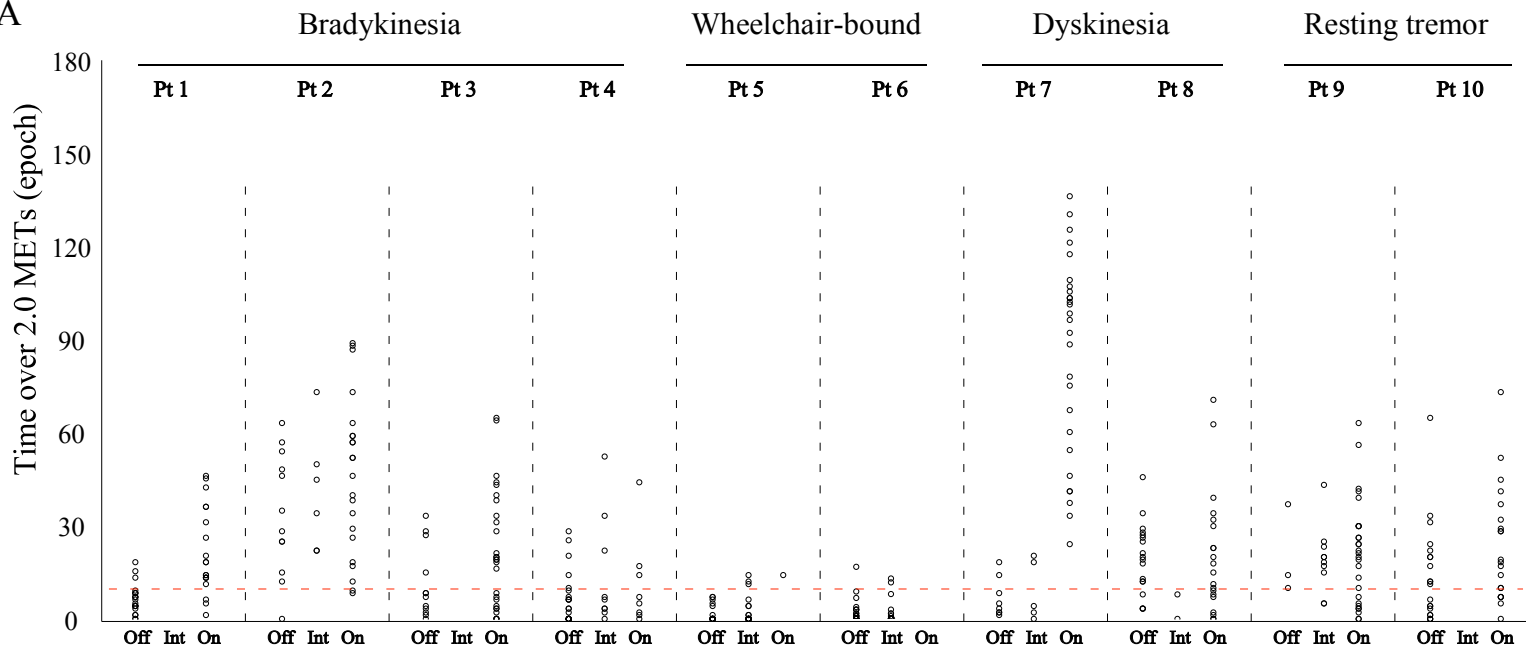

B

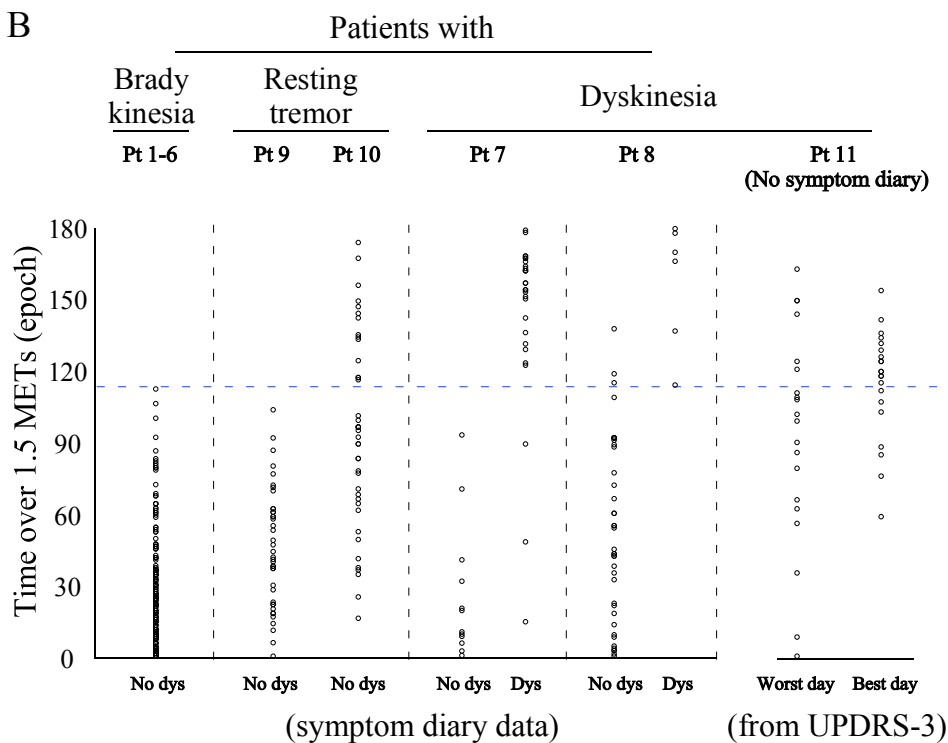

Supplement: Supplementary file 2 — Additional file 2: Figure S2. Dot-plots of “time over 1.5 and 2 METs” and application of their cut-off values. Int, intermediate; Pt, patient. (A) Data were obtained from 10 patients, with 392 segments (dots). As a candidate for a cut-off value of “time over 2.0 METs”, 10 (epochs) were considered (red dashed line). (B) Data were obtained from 10 patients (six patients with bradykinesia, two patients with resting tremor, and two patients with dyskinesia), with 398 segments (dots). We considered 114 (epochs) as a candidate for the cut-off value of “time over 1.5 METs” (blue dashed line). Supplementary fig. 2B showed that patients with resting tremor also had values over the cut-off values. The application of the cut-off value for patient 11 (a patient with dyskinesia, who could not record a symptom diary) suggests increased dyskinesia time on the best day. [file 12883_2020_1896_MOESM2_ESM.pdf]
