# Supplementary material for: The relationships between three-axis accelerometer measures of physical activity and motor symptoms in patients with Parkinson’s disease: a single-center pilot study
Source: BMC Neurol. 2020 Sep 10;20:340. doi: 10.1186/s12883-020-01896-w (PMC7488269; doi:10.1186/s12883-020-01896-w)
Supplement: Supplementary file 3 — Additional file 3: Figure S3. Production process and criteria of activity patterns to distinguish “On” from “Non-On”. (A) ROC curve of “time over 2 METs” and “peaks over 2.5 METs”. To develop the criteria, we focused on the better predictors “time over 2 METs” and “peaks over 2.5 METs” for “On” (Tables 2 and 3). The cut-off values of these measures determined by the smallest distance to the top-left corner of the ROC box were 10 and 3 epochs, respectively. From these cut-off values, we calculated four (> 10 epochs/3 epochs = 3.3) or more consecutive epochs over 2 METs in Criterion I and four or more peaks greater than 2.5 METs in Criterion II. (B) The percentage and number of peaks over 4 METs on the worst and best days. The percentage appeared to be low (mean percentage; 3.1 [1.2–6.2] and 2.0 [0.81–5.3], p = 0.12;mean number; 13.7 [7–28] and 12.6 [8–34], p = 0.51), and a relatively higher percentage and larger number were observed on the worst day, suggesting that some PD patients on the worst day tended to have more peaks over 4 METs. On the basis of these results, we developed the criteria for exclusion of peaks ≥4 METs. (C) Criteria for activity patterns to distinguish “On” from “Non-On”. The definitions of the criteria for “On”, “Non-On” and “tremor (resting tremor)” . The criteria for dyskinesia are also described for reference. Regarding “tremor” (the sub-score of resting tremor in UPDRS-3 ≥ 2), we focused on “time over 1.5 METs”. A cut-off value of 90 epochs was determined, because patients with resting tremor showed milder elevation of baseline of METs compared with dyskinesia (reference) and almost all patients with bradykinesia did not have values over 90 epochs (Supplementary Fig. 1B). “Tremor” was classified as “Non-On”, even when patients met the criteria for “On”. [file 12883_2020_1896_MOESM3_ESM.pdf]

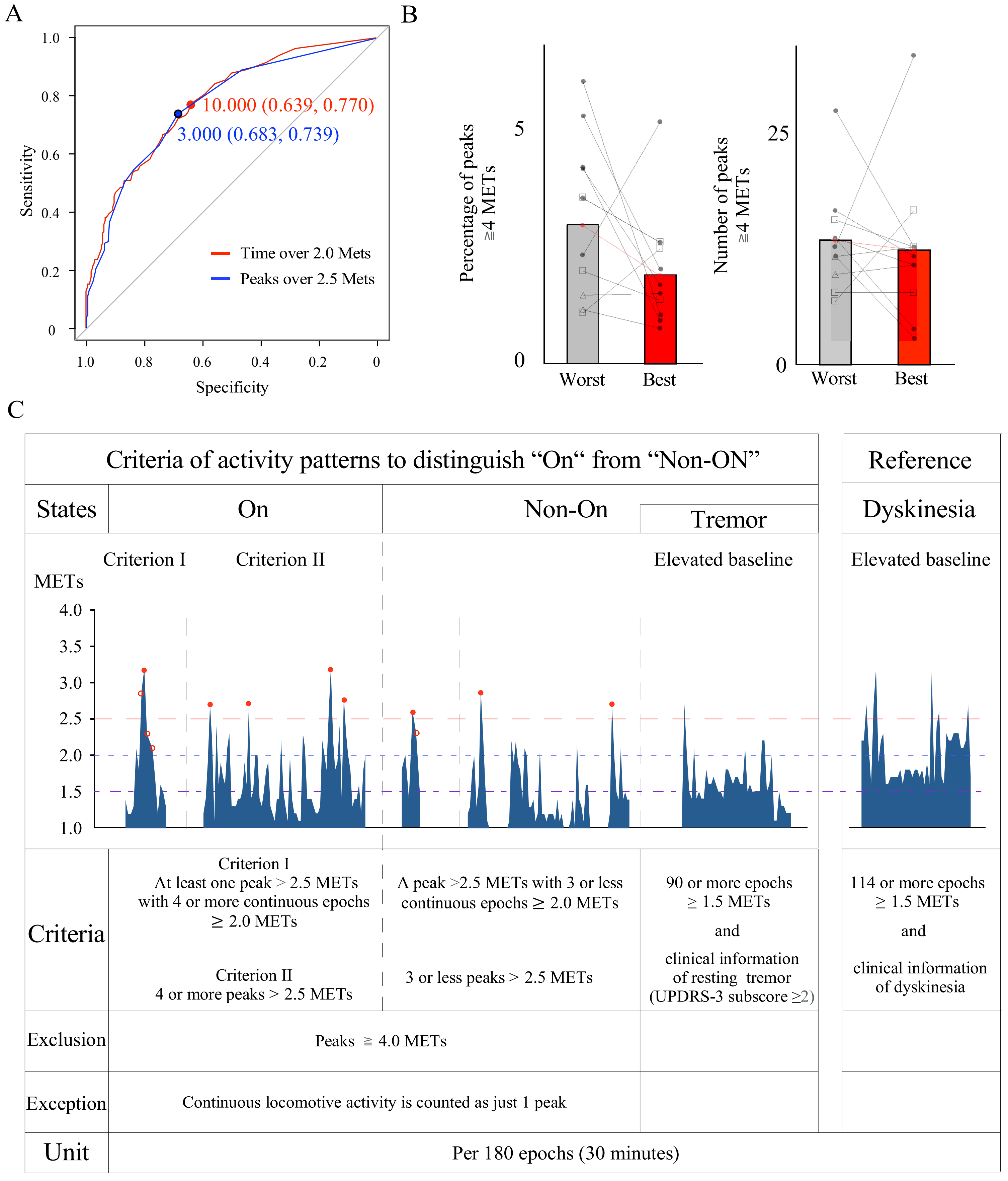
A
1.0
0.8
0.6
0.4
0.2
0
Sensitivity
10.000 (0.639, 0.770)
3.000 (0.683, 0.739)
Time over 2.0 Mets
Peaks over 2.5 Mets
1.0
0.8
0.6
0.4
0.2
0
Specificity
B
Percentage of peaks ≧4 METs
5
0
Worst
Best
Number of peaks ≧4 METs
25
0
Worst
Best
C
Criteria of activity patterns to distinguish “On“ from “Non-ON”
Reference
States
On
Non-On
Tremor
Dyskinesia
Criterion I
Criterion II
Elevated baseline
Elevated baseline
METs
4.0
3.5
3.0
2.5
2.0
1.5
1.0
Criteria
Criterion I
At least one peak > 2.5 METs with 4 or more continuous epochs ≥ 2.0 METs
Criterion II
4 or more peaks > 2.5 METs
A peak >2.5 METs with 3 or less continuous epochs ≥ 2.0 METs
3 or less peaks > 2.5 METs
90 or more epochs ≥ 1.5 METs
and
clinical information of resting tremor (UPDRS-3 subscore ≥2)
114 or more epochs ≥ 1.5 METs
and
clinical information of dyskinesia
Exclusion
Peaks ≧ 4.0 METs
Exception
Continuous locomotive activity is counted as just 1 peak
Unit
Per 180 epochs (30 minutes)
